# *Allium kazim-kosei*, a New Species (*A*. sect. *Codonoprasum*, Amaryllidaceae) from Central Anatolia (Türkiye)

**DOI:** 10.3390/life16050852

**Published:** 2026-05-20

**Authors:** Yavuz Bülent Köse, Mehmet Maruf Balos

**Affiliations:** 1 Department of Pharmaceutical Botany, Faculty of Pharmacy, Anadolu University, 26470 Eskişehir, Türkiye; 2 Department of Pharmaceutical Botany, Faculty of Pharmacy, Harran University, 63050 Şanlıurfa, Türkiye; mbalos@gmail.com

**Keywords:** *Allium*, *Codonoprasum*, new species, Türkiye, gypsum soils, molecular phylogeny, ITS, *trnL*, SEM

## Abstract

*Allium kazim-kosei sp. nov.* (Amaryllidaceae, sect. *Codonoprasum*) is described as a new species from Central Anatolia, Türkiye. The new species is morphologically similar to *A. pseudoflavum* but differs in several diagnostic characters, including bulb structure, scape height, leaf morphology, spathe venation, inflorescence and pedicel dimensions, tepal shape, presence of interstaminal teeth, capsule shape, and seed size. SEM observations reveal distinct micromorphological differences in seed testa ornamentation and pollen exine structure between the two species. Molecular phylogenetic analyses based on nuclear ITS and chloroplast *trnL* intron sequences support the recognition of *A. kazim-kosei* as a distinct species. The Kimura 2-parameter (K2P) genetic distance between *A. kazim-kosei* and *A. pseudoflavum* (8.54% for ITS) is considerably higher than typical interspecific divergences within sect. *Codonoprasum*. In the Maximum Likelihood phylogenetic tree, the two species form well-separated sister branches with high bootstrap support. The species is known only from gypseous soils around Kavuncu village (Eskişehir province, Türkiye), with an estimated Area of Occupancy (AOO) of 8 km^2^ and Extent of Occurrence (EOO) of 45 km^2^. Based on IUCN criteria, *A. kazim-kosei* is assessed as Endangered (EN) [B1ab(iii) + B2ab(iii)]. This discovery increases the total number of *Allium* species in Türkiye to 239 and the number of sect. *Codonoprasum* taxa to 74. The molecular results are fully congruent with the macro- and micromorphological characters, providing robust multi-evidence support for the recognition of the new species.

## 1. Introduction

The genus *Allium* L. is the largest among Amaryllidaceae, comprising approximately 1078 species distributed throughout the Northern Hemisphere, with primary diversity centers in the Mediterranean and Southwest/Central Asia [1,2,3,4]. *Allium* species share several common characteristics, including bulbs with tough tunics, terminal umbellate inflorescences, and capsules that produce 1–2 seeds per locule [5,6].

*Allium* sect. *Codonoprasum* Rchb. is a group of perennial onions occurring mainly in Central Asia, Southwest Asia, and the Mediterranean Basin [7]. The section is characterized by long, narrow leaves, an obvious spathe, and bell or globular flowers [6,8]. Seed microsculpturing has also been shown to have taxonomic relevance within the section [9].

Molecular studies using nuclear ITS sequences have confirmed the monophyly of sect. *Codonoprasum* and provided information on its phylogenetic position within *Allium* [10]. However, ITS sometimes does not provide enough resolution to distinguish closely related species within the section [9]. Therefore, recent phylogenetic studies have used combinations of nuclear and chloroplast markers [9,10]. Salmeri et al. [9] combined ITS and trnH-psbA data to investigate *A. paniculatum* and recovered two clades within the section. Recently, Duchoslav et al. [10] used three genomic loci (nrITS, *trnH-psbA*, and *trnL-ndhJ*) to analyze 48 taxa (approximately 30% of the section’s diversity) and identified five major lineages (clades I–V), revealing that traditional subsectional taxonomy is largely incongruent with phylogenetic patterns.

Chloroplast markers, particularly the *trnL* intron, have proven useful in *Allium* systematics, evolving rapidly and providing good variation for species-level phylogenetic analyses [11,12,13]. Numerous studies have successfully used the *trnL* intron to delimit species and reconstruct phylogenies of *Allium*, including sect. *Codonoprasum* [11,12,13,14,15]. The combined use of ITS and *trnL* markers enables recognition of incongruences indicating hybridization or incomplete lineage sorting [1,15]. Genetic distance calculations have become standard criteria for species delimitation in *Allium* [15,16]. The Kimura 2-parameter (K2P) model [17] is widely accepted for quantifying evolutionary divergence. Interspecific genetic distances within sect. *Codonoprasum* average from 0.5% to 5% for ITS, with values above this range indicating well-differentiated species [11,15]. The Maximum Likelihood (ML) method with bootstrap resampling [18] and the Tamura-Nei (TN93) model [19] are commonly used for phylogenetic reconstruction in *Allium* [11,19].

Recent identification of new species in sect. *Codonoprasum* from Türkiye and adjacent areas has relied on integrative taxonomic methodologies combining morphology, micromorphology, and molecular phylogenetics [11,12,14,15,20,21,22]. Examples include *A. gilanense* Bagheri & R.M.Fritsch (Iran) [15], *A. serpenticola* Eker [14], *A. euphraticum* Sonay, Balos & Cakilcioglu [11], *A. kubeysdaghense* Sonay, Gül, Balos & Bagci [12], *A. sadikerikii* Eker & Özüdoğru [20], and *A. turcicum* subsp. *fusciflorum* Çeçen, Akan, Geçit & Balos [13]. These studies have shown that Anatolia, especially Central and Eastern Anatolia, is a global hotspot for cryptic and narrow endemic diversity in sect. *Codonoprasum* [11,13,14,20].

Nuclear ITS regions support the monophyly of the group, but plastid regions alone do not provide sufficient resolution [1,9]. The taxonomy of sect. *Codonoprasum* has always been problematic, but recent revisions are slowly clarifying its limits [6]. Taxa in this section are predominantly found in arid steppes, serpentine soils, and rocky habitats, with particularly high speciation rates in Anatolia [5,11,14].

Türkiye currently has 238 species of *Allium* [21]. Sect. *Codonoprasum* is the second-largest section in Türkiye, containing 73 taxa (43 endemics) [11,21]. Following the description of *A. kazim-kosei*, the total number of *Allium* species in Türkiye will increase to 239, and the number of sect. *Codonoprasum* taxa to 74.

During field studies in Central Anatolia, we encountered a distinctive *Allium* population growing on gypseous soils around Kavuncu village (Günyüzü district, Eskişehir province). This paper aims to provide a comprehensive morphological description of this new species, compare it with its closest relative *A. pseudoflavum*, present micromorphological (SEM) data, conduct molecular phylogenetic analyses using ITS and *trnL* sequences, and assess its conservation status. Consequently, we formally propose *Allium kazim-kosei* as a new species in sect. *Codonoprasum*.

## 2. Materials and Methods

### 2.1. Plant Material and Morphological Studies

The specimens on which the description of the new taxon is based were collected from Günyüzü district of Eskişehir province, Türkiye, in June 2025. After drying, approximately 10 specimens collected from three localities were deposited in HUEH (Harran University Faculty of Pharmacy Herbarium) and ESSE (Anadolu University, Faculty of Pharmacy Herbarium). After detailed morphological observations on living material, we concluded that the specimens represent a previously undescribed species. *Allium kazim-kosei* is here compared with *A. pseudoflavum* Vved., its closest morphological relative. The specimens on which the description of the new taxon is based were collected from Günyüzü district of Eskişehir province, Türkiye, in June 2025. A total of 45 specimens were collected from localities within the plant natural range, covering the entire known distribution of the species. For each species, 20 individuals were measured for quantitative morphological characters. For the identification and comparison of the new species, we consulted primary regional floras including Mouterde [23] for Lebanon, and other key works on *Allium* from adjacent territories: Türkiye [5,24,25], Iran [26], Iraq [27], Syria and Palestine [28], as well as historical references such as Post [29] and Boissier [30]. Additional literature included Cowley et al. [31], Bagheri et al. [15], Balos [32,33], Balos et al. [34], Özdöl et al. [35], Balos & Geçit [36,37], Koçyiğit et al. [38,39], Jang et al. [40], Koçyiğit et al. [41], Eker [14,16], Yıldırım et al. [42], Sonay et al. [12,43], Çeçen et al. [13], Özüdoğru & Eker [20] and Eker [21].

### 2.2. DNA Extraction, Amplification and Sequencing

Total genomic DNA was extracted from silica gel-dried leaf material of 20 individuals (10 per species) using the EurX GeneMATRIX Plant & Fungi DNA isolation kit (Gdańsk-Poland) following the manufacturer’s protocol. Two DNA regions were amplified: (1) the nuclear ribosomal internal transcribed spacer (ITS) including ITS1, 5.8S, and ITS2, using primers ITS1 and ITS4 [44]; and (2) the chloroplast *trnL* (UAA) intron using primers c and d [45]. PCR reactions were performed in a 35 μL volume containing 6 μL 2 × Taq Plus PCR MasterMix (Solis Biodyne FIREPol^®^ DNA Polymerase, Tartu-Estonia), 3 μL DNA template, 1.5 mM MgCl_2_, 0.3 μM of each primer, 0.2 mM dNTP mix, and PCR-grade water. The thermal cycling profile consisted of initial denaturation at 95 °C for 5 min, followed by 30 cycles of denaturation at 95 °C for 45 s, annealing at 57 °C for 45 s, and extension at 72 °C for 60 s, with a final extension at 72 °C for 5 min. PCR products were visualized on 1% agarose gel electrophoresis and sequenced bidirectionally by BM Labosis (Türkiye).

### 2.3. Sequence Alignment and Dataset Assembly

Chromatograms were edited and assembled using BioEdit v.7.2 [46]. Newly generated sequences of A. *kazim-kosei* and *A. pseudoflavum* were submitted to GenBank (accession numbers provided in Appendix A). Additional sequences of related *Allium* species from sect. *Codonoprasum* and allied sections were retrieved from GenBank. Multiple sequence alignments were performed separately for ITS and *trnL* datasets using ClustalW [47] implemented in MEGA 12.0 [48], followed by manual adjustment. The final concatenated alignment comprised 558 positions for ITS (14 taxa) and 632 positions for *trnL* (15 taxa), as indicated in the MEGA output files.

### 2.4. Genetic Distance Analyses

Evolutionary divergence between sequences was estimated using the Kimura 2-parameter (K2P) model [17], which accounts for different transition/transversion mutation rates. Pairwise genetic distances were calculated separately for the ITS datasets using MEGA 12.0 [48]. Pairwise deletion was applied to all ambiguous positions for each sequence pair. The uncorrected p-distance values were also calculated for comparison. Genetic distances between *A. kazim-kosei* and its morphologically closest relative *A. pseudoflavum* were extracted from the distance matrices.

### 2.5. Phylogenetic Tree Reconstruction

Maximum Likelihood (ML) phylogenetic analyses were conducted using MEGA 12.0 [48] based on the ITS (28 taxa, 660 bp after alignment) and *trnL* intron (15 taxa, 632 bp) datasets. For model selection, the Bayesian Information Criterion (BIC) implemented in MEGA was used. For the ITS dataset, the Tamura-Nei (TN93) model [19] with gamma-distributed rate heterogeneity (TN93 + G) was identified as the best-fitting model (BIC = 6421.972; lnL = −2939.829). For the *trnL* dataset, the T92 model was selected (BIC = 2954.647; lnL = −1366.425).

Sequence alignments were performed separately for each marker using ClustalW [47] with default parameters: gap opening penalty = 15, gap extension penalty = 6.66, transition weight = 0.5. For gap treatment, pairwise deletion was applied for all phylogenetic analyses, meaning that sites with gaps were removed on a pairwise basis as needed. For the ML tree search, the NNI (Nearest Neighbor Interchange) heuristic method was used with 1000 bootstrap replicates [18]. Bootstrap values (BS) ≥ 70% were considered statistically significant [49]. *A. caesium* Schrenk and *A. ursinum* L. (sect. *Arctoprasum*) were used as outgroup based on previous phylogenies [1].

For the ITS dataset, we compared the newly generated sequences with those of *Allium* species distributed in Türkiye that were deposited in GenBank by our team, as well as with sequences of closely related species belonging to sect. *Codonoprasum* as identified in the phylogeny of Duchoslav et al. [10]. For the *trnL* dataset, comparisons were made with all *Allium* species available in GenBank for which the *trnL* intron region has been sequenced (Appendix A.2).

For the ITS dataset, pairwise K2P distances among species of sect. *Codonoprasum* ranged from 0.0036 to 0.1619. The genetic distance between *A. kazim-kosei* and *A. pseudoflavum* was calculated as 0.0854 (8.54%) for ITS. These values are considerably higher than typical interspecific divergences within *Allium* sect. *Codonoprasum*, strongly supporting the recognition of *A. kazim-kosei* as a distinct species.

### 2.6. Scanning Electron Microscopy (SEM)

Seed and pollen micromorphology of *A. kazim-kosei* and *A. pseudoflavum* were examined using scanning electron microscopy. For each species, 10 seeds and 20 pollen grains were examined. Specimens were collected from the holotype of *A. kazim-kosei* (ESSE 16430) and the isotype of *A. pseudoflavum* (HUEH M. Balos 5650). Samples were mounted on aluminum stubs using double-sided carbon tape and sputter-coated with 15 nm of gold-palladium using a Quorum Q150T ES sputter coater. Observations were performed using a Zeiss EVO LS10 scanning electron microscope at an accelerating voltage of 10 kV. Measurements (seed size, papilla dimensions, pollen size, and exine lumen diameter) were taken using Zeiss SmartSEM V5.05 software. To ensure statistical reliability, all measurements were replicated across three independent individuals per species, with 10 replicate measurements per structure per individual, and mean values were calculated.

## 3. Results

**Figure 1:**
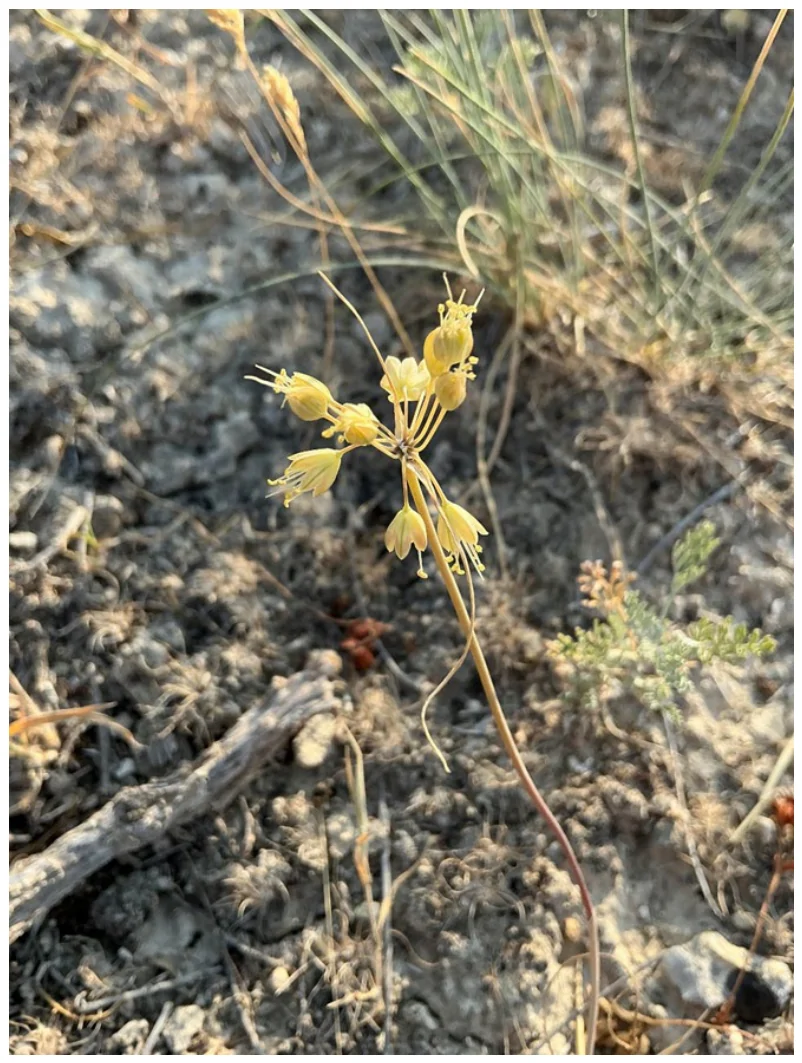
*Allium kazim-kosei*.—Habit at the type locality.

**Figure 2:**
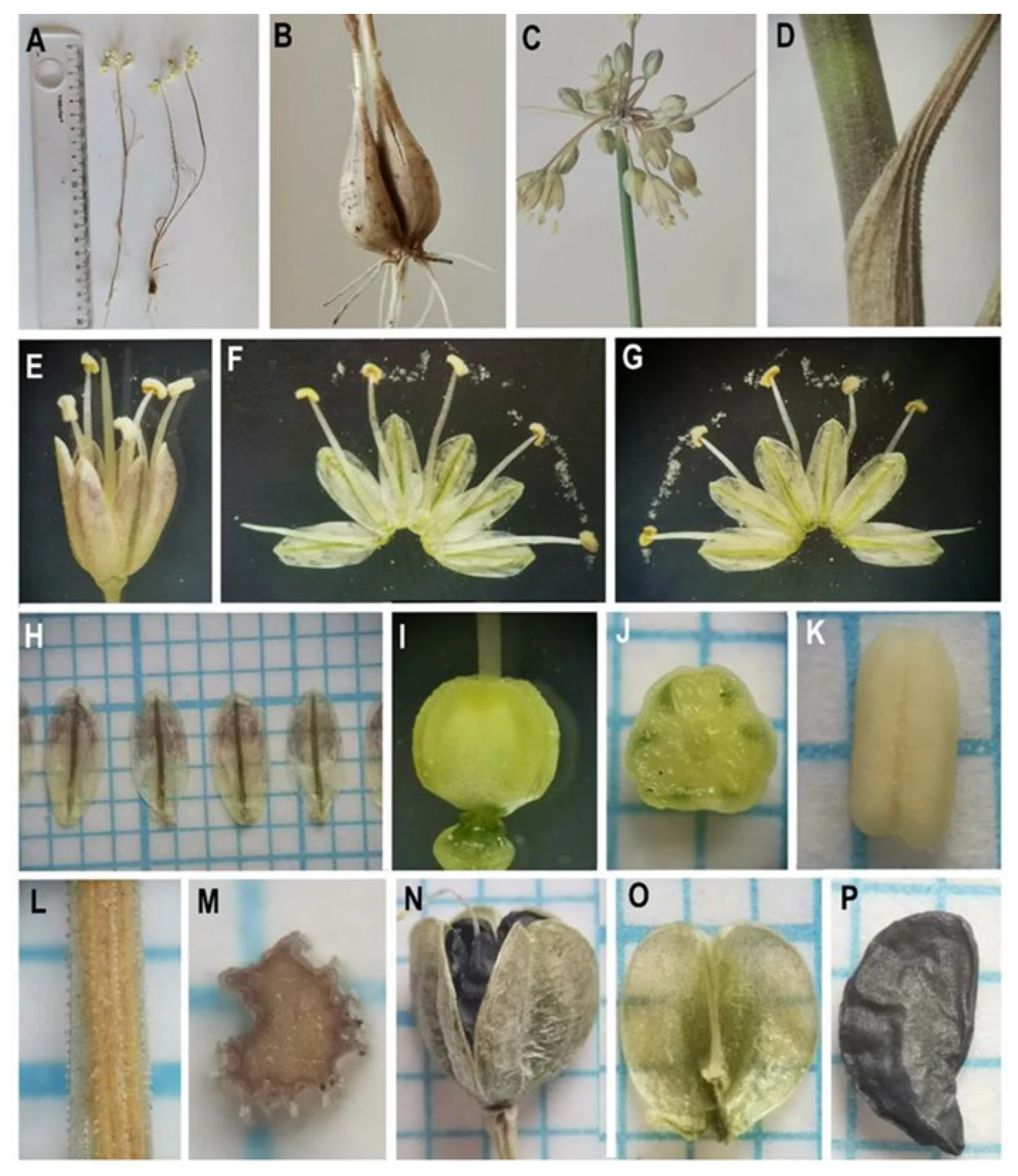
*Allium kazim-kosei*. (**A**): Habit. (**B**): Bulb. (**C**): Inflorescences. (**D**): Leaf sheaths on stem. (**E**): Flower. (**F**): Inner surface of open perigon. (**G**): Outer and inner tepal. (**H**): Tepals. (**I**): Ovary. (**J**): Ovary cross-section. (**K**): Anther. (**L**): Leaves. (**M**). Leaves cross-section. (**N**): Capsule. (**O**): Capsule valves. (**P**): Seed (From holotype, ESSE 16430).

**Figure 3:**
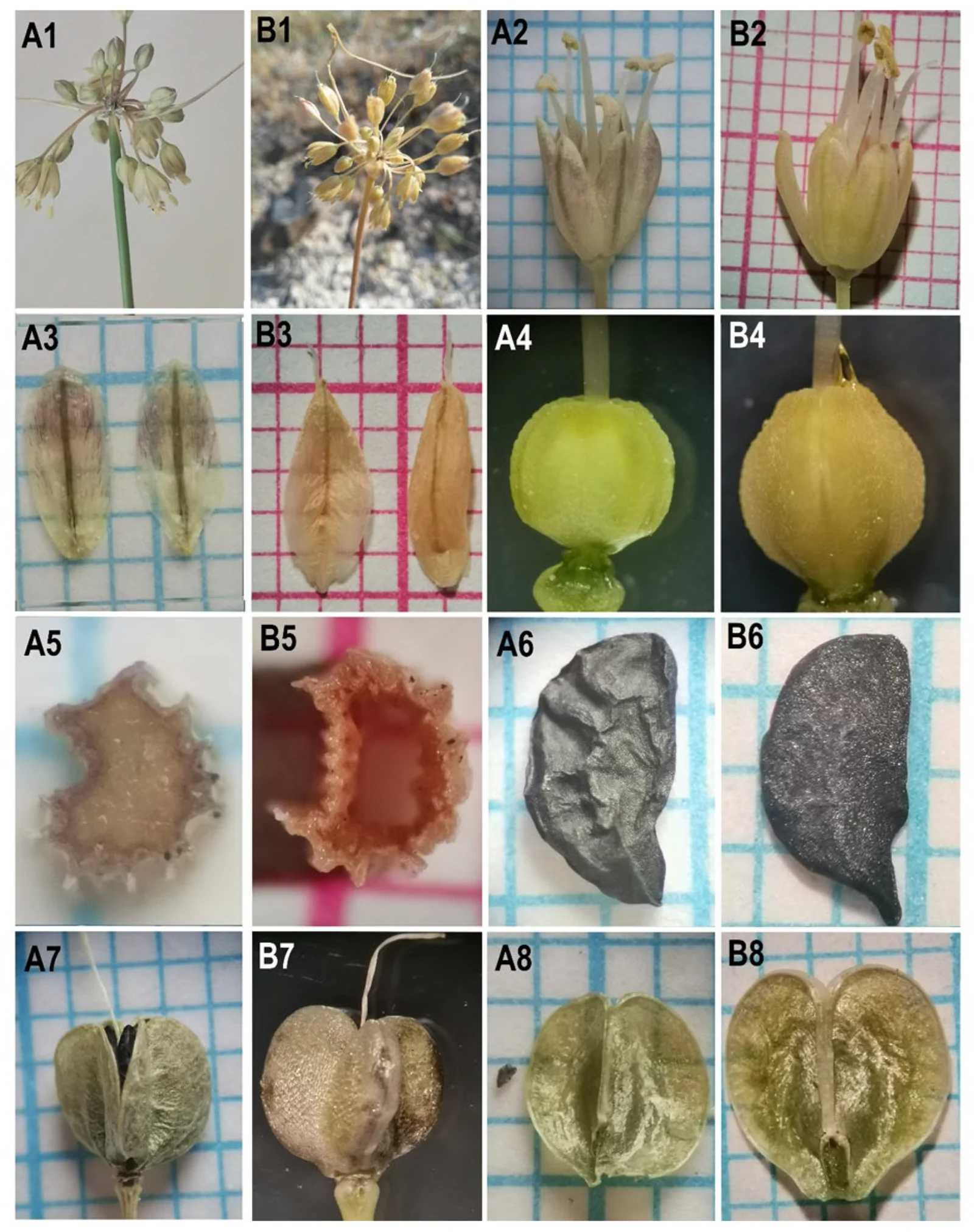
Morphological comparison of (*A. kazim-kosei*: (**A1**–**A8**), from the holotype) and *A. pseudoflavum* (**B1**–**B8**). (**A1**,**B1**): Inflorescences. (**A2**,**B2**): Perigon. (**A3**,**B3**): Tepals. (**A4**,**B4**): Ovary. (**A5**,**B5**): Leaf cross-section. (**A6**,**B6**): Seed. (**A7**,**B7**): Capsule. (**A8**,**B8**): Capsule valves.

*Allium kazim-kosei sp. nov.* Köse & Balos (Figure 1, Figure 2 and Figure 3).

Type: Türkiye, Günyüzü district of Eskişehir province, around Kavuncu village, 39.40830° N, 31.90574° E, gypseous soils, 886 m a.s.l., 12 June 2025, Y.B. Köse (holotype ESSE 16430; isotype HUEH M. Balos 5650).

Diagnosis: *Allium kazim-kosei* differs from *A. pseudoflavum* by its smaller bulbs (1–1.2 × 0.4–0.6 cm vs. 1.5–2 × 0.5–1.5 cm), creamy-white to light brown outer tunics with a shorter neck (0.5–1 cm vs. 1.5–4 cm), shorter scape (12–20 cm vs. 20 × 50 cm), shorter solid leaves (6–15 cm vs. 20–30 cm, hollow), shorter and fewer-veined spathe valves (3–5 vs. 8–10 veins), smaller inflorescences with fewer flowers (7–18 vs. 10–25), shorter pedicels (0.5–1.2 cm vs. 1.5–2.5 cm), smaller perigon with equal tepals (vs unequal), whitish filaments with present interstaminal teeth (vs absent), a narrower and smaller ovary (1.5–1.8 × 1.5–1.9 mm vs. 1.5–2 × 2–2.5 mm), ovoid capsule (vs globose), and smaller seeds (2.8–3 × 1.5–2.5 mm vs. 3.5–4 × 2–2.5 mm) (Table 1).

Description: Bulbous perennial. Bulb oblong-ovoid, usually growing in pairs, each bulb produces one scape, 10–12 × 4–6 mm; outer tunics creamy-white to light brownish; inner tunic brownish, with distinct parallel veins, coriaceous; 0.5–1 cm neck formed on the stem. Scape 12–20 cm × 1 mm, cylindrical, glabrous, green, sometimes purplish at base, covered by leaf sheaths at 1/2–2/3 of its length. Leaves 2–3 (–4), linear-filiform, 6–15 cm × 0.5–0.8 mm, semicylindrical, lower sheaths and lamina scabrid, solid, shorter than the scape. Spathe with two unequal, linear-lanceolate valves; the longer valve 4–4.5 (−10) × 0.2–0.3 cm, with 3–5 veins, exceeds the umbrella; the shorter valve 2.5–3.5 × 0.15–0.20 cm, with 2–5 veins. Inflorescence a lax, hemispherical umbel, 2–2.5 cm in diameter, 7–18 flowered. Pedicels are the same color as the perigon, unequal, 5–12 mm, bracted at the base. Perigone campanulate, 4–4.5 × 3.5–4 mm; tepals equal, 4.2–4.5 × 1.8–2 mm, mucronate, elliptic to ovate; outer tepals boat-shaped, upper edges of inner tepals curved inwards; pale yellow, sometimes with brown markings on the upper half, with prominent green veins. Filaments exserted, filiform, off-white, 4.5–5.5 mm; with short interstaminal teeth; annulus 0.75–0.9 mm; anthers oblong, yellow-cream, c. 1 mm. Ovary yellow, narrowly globose, with small stalk, 1.50–1.80 × 1.50–1.90 mm; surface reticulate, margins clearly tuberculous; style filiform, 5–5.5 mm, exerted to the apex of the tepals. Capsule ovoid, c. 3.9–4 × 3–3.5 mm, 3-locular; valves ovoid, 2.5–3.5 × 3–3.5 mm emarginate at apex. Seeds black, flat, c. 2.83 × 1.5–2.5 mm.

Phenology: Flowering in June; fruiting from late June to July.

Distribution and Habitat: *A. kazim-kosei* is native to the gipsum area around Kavuncu village, Günyüzü in Eskişehir. Associated species include *Thymus leucostomus* Hausskn. & Velen., *Minuartia anatolica* (Boiss.) Woronow, *Cynanchica bornmuelleri* (Velen. ex Bornm.) P.Caputo & Del Guacchio., *Astragalus karamasicus* Boiss. & Balansa *Lomelosia argentea* (L.) Greuter & Burdet., *Bassia prostrata* (L.) Beck, *Consolida raveiyi* (Boiss.) Schröd., *Gypsophila heteropoda* Freyn, *Anthemis cretica* L., *Gypsophila eriocalyx* Boiss., *Alyssum sibiricum* Willd., *Festuca valesiaca* Schleich. ex Gaudin, *Centaurea virgata* Lam., *Euphorbia macroclada* Boiss., *Nigella arvensis* L., *Helianthemum ledifolium* (L.) Mill., *Artemisia santonicum* L., *Bromus tomentellus* Boiss., etc.

Etymology: The species is named in honor of the author’s (Yavuz Bülent Köse) father, Kazım Köse.

Conservation status: The species has an extremely limited geographic range. According to field surveys and distribution modeling, the estimated Area of Occupancy (AOO) is 8 km^2^ and the Extent of Occurrence (EOO) is 45 km^2^, both of which fall within the thresholds for Endangered (AOO < 500 km^2^; EOO < 5000 km^2^). Based on the species’ restricted range and its occurrence at a single location, a continuing decline is inferred under IUCN Criterion B [50]. Accordingly, based on the IUCN Red List Categories and Criteria 50]), the species is assessed as “Endangered” (EN) [B1ab(iii) + B2ab(iii)].

Seed micromorphology: The seed and pollen micromorphology of *A. kazim-kosei* and *A. pseudoflavum* were examined by SEM. Clear micromorphological differences between the two taxa were recorded in both seed testa ornamentation and pollen exine structure.

**Figure 4:**
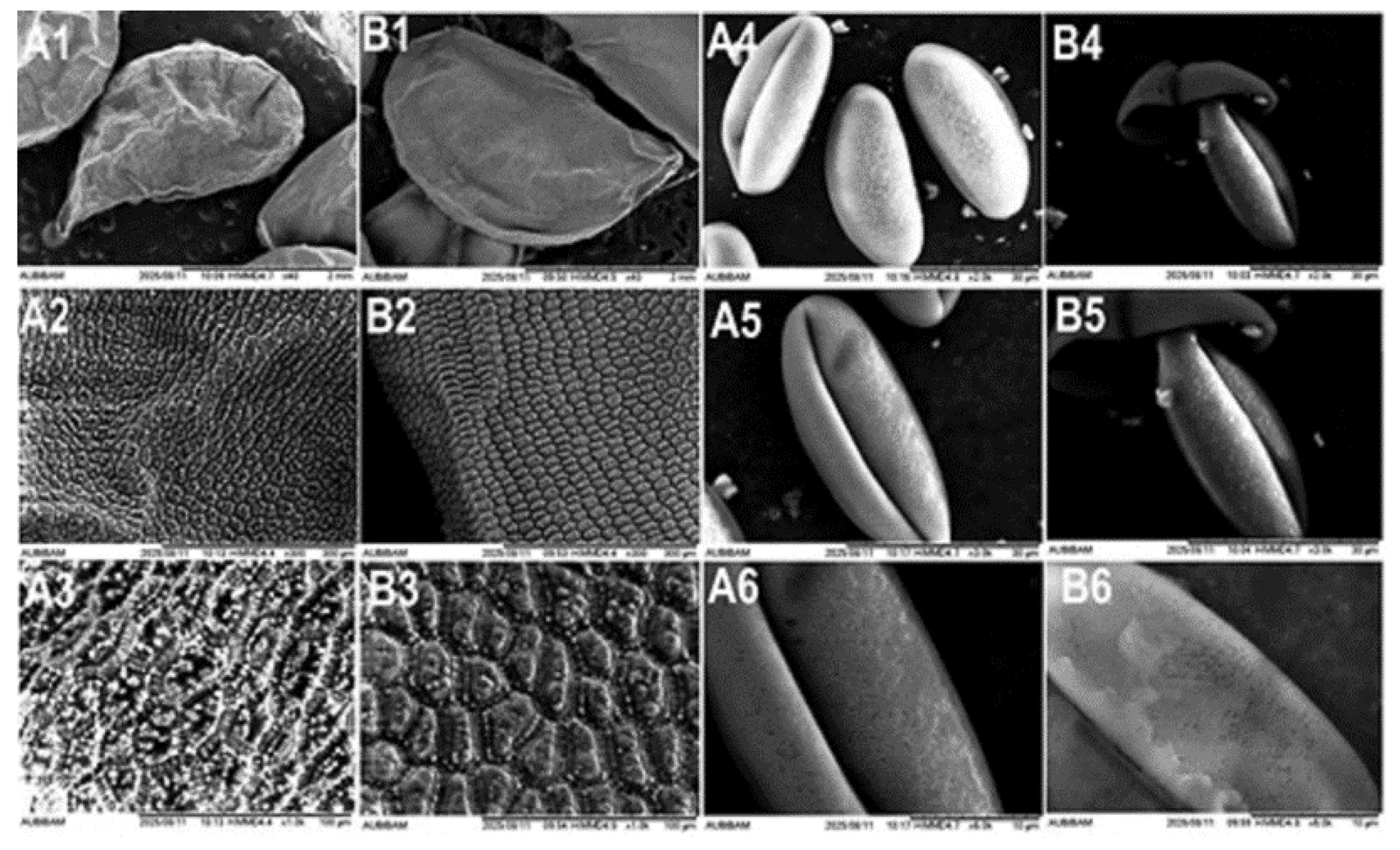
Scanning electron micrographs of seed and pollen micromorphology of *A. kazim-kosei* (**A1**–**A6**) and *A.pseudoflavum* (**B1**–**B6**). (**A1**): General view of seed (×40; scale bar = 2 mm). (**A2**): Seed testa at medium magnification showing papilla distribution (×300; scale bar = 300 µm). (**A3**): High-magnification view of papillae on testa (×1.0k; scale bar = 100 µm). (**B1**): General view seed (×40; scale bar = 2 mm). (**B2**): Seed testa at medium magnification showing denser, smaller papillae (×300; scale bar = 300 µm). (**B3**): High-magnification view of papillae on testa (×1.0k; scale bar = 100 µm). Pollen panels ((**A4**–**A6**); (**B4**–**B6**)). (**A4**): General view A pollen grains (×2.0k; scale bar = 30 µm) (**A5**): Pollen exine at medium magnification showing microreticulate pattern for A (×3.0k; scale bar = 30 µm). (**A6**): High-magnification detail exine (lumina and muri) (×6.0k; scale bar = 10 µm). (**B4**): General view of pollen grains (×2.0k; scale bar = 30 µm). (**B5**): Pollen exine at medium magnification showing denser exine structure for B (×3.0k; scale bar = 30 µm). (**B6**): High-magnification detail of B exine (×6.0k; scale bar = 10 µm).

*A. kazim-kosei* seeds are elliptic-oval, relatively larger, measuring 2.8–3.5 mm in length and 1.5–2.0 mm in width (Figure 4(A1), Table 2). Testa ornamentation is regularly papillate with moderately large, dome-shaped papillae that are arranged in a regular pattern. Measured papilla dimensions: height 15–20 µm, width 20–30 µm (Figure 4(A2,A3), Table 2). Anticlinal walls are raised and distinct; periclinal walls slightly convex, producing a somewhat “bubbled” appearance across the testa.

*A. pseudoflavum* seeds are slightly smaller: 2.5–3.0 mm long and 1.5–1.8 mm wide (Figure 4(B1), Table 2). The testa is densely papillate but with smaller and more compact papillae compared to *A. kazim-kosei* papilla measurements: height 10–15 µm, width 15–25 µm (Figure 4(B2,B3), Table 2). Papillae are more closely packed, giving a finer, more even texture; anticlinal walls are distinct but periclinal surfaces are less convex than in *A. kazim-kosei*.

Pollen micromorphology: *Allium kazim-kosei* (Figure 4(A4–A6), Table 2). Pollen grains are trizonocolpate, prolate-elliptic, larger than in *A. pseudoflavum*: P (polar axis) 42–47 µm, E (equatorial axis) 23–27 µm (Figure 4(A4)). Exine is microreticulate with lumina of ~0.5–1.0 µm and moderately thick muri (Figure 4(A5,A6), Table 2). Colpi are long, exine thickness moderate; overall exine appearance is moderately open compared to *A. pseudoflavum*.

*A. pseudoflavum* pollen grains are slightly smaller: P 38–43 µm, E 21–25 µm (Figure 4(B4)). Exine is also microreticulate but lumina are smaller (~0.3–0.8 µm) and muri are thicker and more prominent (Figure 4(B5,B6), Table 2). Apertures (colpi) appear slightly narrower.

*A. kazim-kosei* shows larger pollen grains with larger lumina and moderately thick muri, whereas *A. pseudoflavum* has smaller pollen with denser exine (smaller lumina and thicker muri). These consistent differences in pollen size and exine microstructure support taxonomic separation.

**Figure 5:**
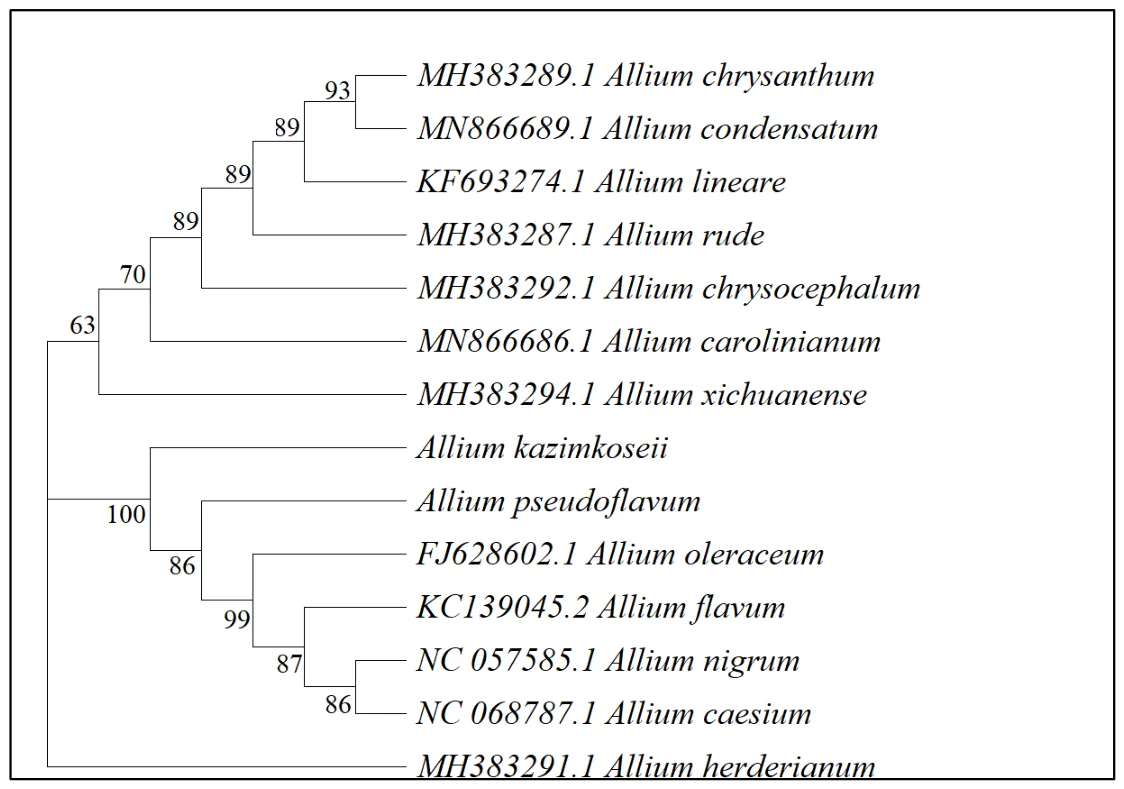
Maximum Likelihood (ML) tree inferred from *trnL* intron sequences (15 taxa, 632 bp). Bootstrap values (1000 replicates) are indicated above the branches. *Allium caesium* served as outgroup.

Phylogenetic analyses of *trnL* intron dataset (15 taxa, 632 aligned positions) using the Tamura-Nei (TN93 + G) model [19] produced a well-resolved tree with high bootstrap support (Figure 5). In particular, *A. kazim-kosei* and *A. pseudoflavum* presented as adjacent yet distinctly separated sister taxa. The clade containing *A. kazim-kosei* was supported with a bootstrap value of 88% whereas *A. pseudoflavum* presented a bootstrap support of 98%. Other members of the sect. *Codonoprasum*, including *A. oleraceum* L., *A. flavum* L., *A. lineare* L., *A. condensatum* Turcz., *A. carolinianum* DC., *A. rude* J.M.Xu, *A. chrysocephalum* Regel, *A. chrysanthum* Regel, and *A. xichuanense* J.M.Xu, created distinct, well-supported clades without clustering with respect to *A. kazim-kosei*. *A. caesium* served as an outgroup and separated from all sect. *Codonoprasum* taxa [1].

**Figure 6:**
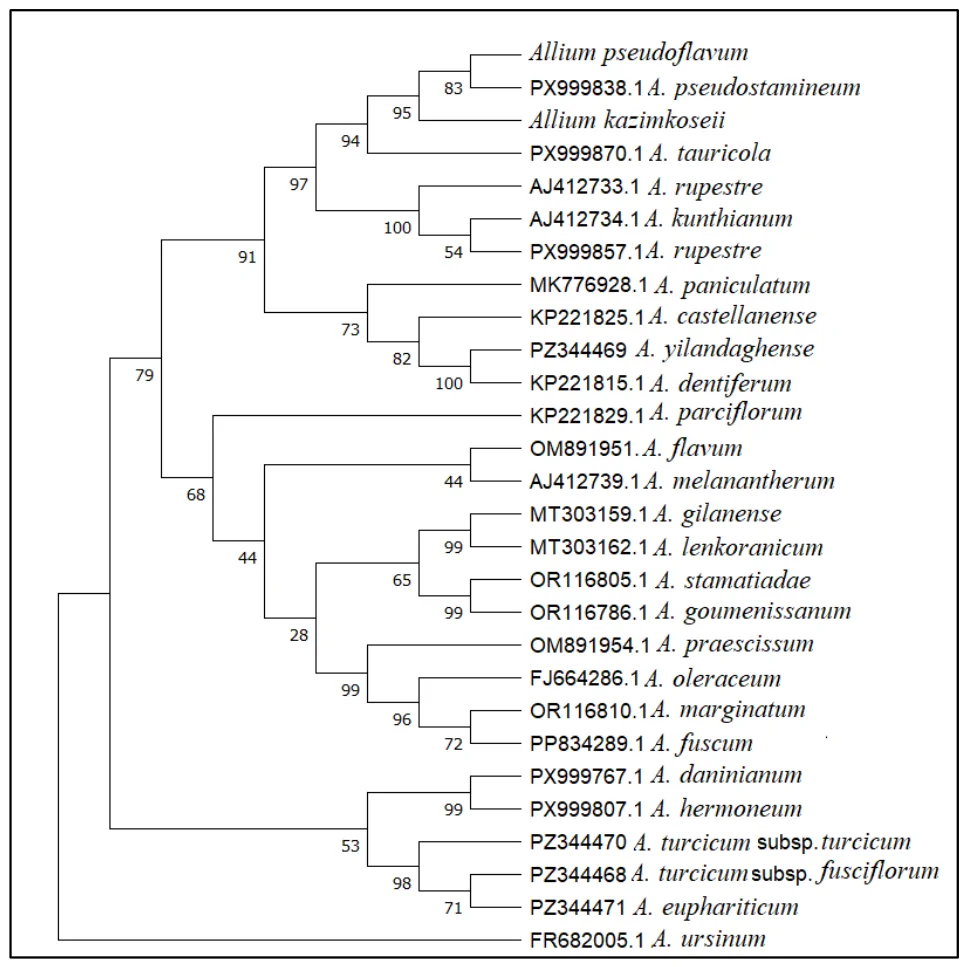
Maximum Likelihood (ML) tree inferred from ITS sequences (28 taxa, 660 bp). Bootstrap values (1000 replicates) are indicated above the branches. *Allium ursinum* served as outgroup.

Maximum Likelihood (ML) phylogenetic analysis was performed on the nuclear ITS dataset comprising 28 taxa and 660 aligned positions with 1000 bootstrap replicates (Figure 6). The resulting tree is well-resolved. *A. kazim*-*kosei* and *A. pseudoflavum* form two adjacent sister branches, with bootstrap support of 100% for *A. kazim-kosei* and 98% for *A. pseudoflavum.* The outgroup *A. ursinum* is placed at the base, consistent with previous phylogenies of *Allium* [1]. The remaining members of sect. *Codonoprasum* included in the analysis form distinct clades and do not cluster with *A. kazim*-*kosei.* These include *A. pseudostamineum* Kollmann & Shmida, *A. tauricola* Boiss., *A. rupestre* Steven, *A. kunthianum* Vved., *A. paniculatum* L., *A. castellanense* Brullo, Guglielmo, Pavone & Salmeri, *A. yilandaghense* Balos & Sonay, *A. dentiferum* Webb & Berthel., *A. parciflorum* Vved., *A. flavum* L., *A. melanantherum* Pančić, *A. gilanense* Bagheri & R.M.Fritsch, *A. lenkoranicum* Miscz. ex Grossh., *A. stamatiae* Trigas, *A. goumenissanum* Ioannidis & Tzanoud., *A. praescissum* Rchb., *A. oleraceum* L., *A. marginatum* Janka, *A. fuscum* Waldst. & Kit., *A. daninianum* Brullo, Pavone & Salmeri, *A. hermoneum* (Kollmann & Shmida) Brullo, Guglielmo, Pavone & Salmeri, *A. turcicum* subsp. *turcicum* Özhatay & Koçyiğit, *A. turcicum* subsp. *fusciflorum* Çeçen, Akan, Geçit, Sonay & Balos, and *A. euphraticum* Sonay, Balos & Çakılcıoğlu. None of these taxa cluster with *A. kazim-kosei*.

Pairwise genetic distances were calculated separately for the ITS using the Kimura 2-parameter (K2P) model [17] (Table A1). For the 13-taxon, 558-position ITS dataset, the genetic distance between *A. kazim-kosei* and *A. pseudoflavum* was 0.0854 (8.54%); for the 15-taxon [1]. *A. kazim-kosei*’s genetic distances from members of sect. *Codonoprasum* were similarly varied: to *A. kunthianum* (0.114), *A. parciflorum* (0.151), *A. tardans* (0.154), *A. gilanense* (0.160), and *A. lenkoranicum* (0.167) over the five taxa. Both ITS and *trnL* intron molecular phylogenetic results are fully congruent with morphological and micromorphological traits and the results indicate that the characters that separate *A. kazim-kosei* from *A. pseudoflavum* include: bulb shape and structure; leaf thickness; spathe venation; number of interstaminal teeth; size and arrangement of the seed’s papillae; and diameter of the pollen’s exine lumen. All these morphological differences exactly correspond to the genetic divergence detected using both nuclear and chloroplast markers.

Appendix A Table A1. For the ITS dataset (14 taxa, 558 positions), the genetic distance between *A. kazim-kosei* and *A. pseudoflavum* was 0.0854 (8.54%).

*Allium kazim-kosei* is currently known from a restricted area on gypseous soils around Kavuncu village, Günyüzü district, Eskişehir province (Central Anatolia, Türkiye), at approximately 886 m a.s.l. The estimated Area of Occupancy (AOO) is 8 km^2^ and the Extent of Occurrence (EOO) is 45 km^2^.

Based on the IUCN Red List Categories and Criteria, *A. kazim-kosei* is assessed as Endangered (EN) under Criteria B1ab(ii,iii) + B2ab(ii,iii). The Extent of Occurrence (EOO) is 45 km^2^ and the Area of Occupancy (AOO) is 8 km^2^, both of which fall within the thresholds for Endangered. The species is known from a restricted range and a continuing decline is inferred in the Area of Occupancy and the quality of its gypseous habitat due to anthropogenic pressures.

## 4. Discussion

The present study describes *A. kazim-kosei* as a new species of *Allium* sect. *Codonoprasum* from the Central Anatolia region of Türkiye. The taxon is identified on the basis of a suite of distinctive morphological characters.

This deep genetic split is directly aligned with and provides an evolutionary context to our extensive morphological findings. While *A. kazim-kosei* is morphologically similar to *A. pseudoflavum*, our extensive comparison reveals a suite of stable and definitive differential characters in various organs. For instance, *A. kazim-kosei* has creamy-white to pale brownish outer bulb tunics and a distinct 0.5–1 cm stem neck, in contrast to the grayish-brown tunics and longer neck (1.5–4 cm) of *A. pseudoflavum*. The leaves also differ; *A. kazim-kosei* has 2–3 (−4) solid leaves shorter than the scape, whereas *A. pseudoflavum* has 2–4 hollow leaves as long as or longer than the scape.

The other important morphological distinctions include the spathe valves, perigone structure, and reproductive organs. The longer spathe valve of *A. kazim-kosei* (4–4.5 (−10) cm with 3–5 veins) is shorter and less veined than that of *A. pseudoflavum* (7–12 cm with 8–10 veins). Most significantly, the short interstaminal teeth found in *A. kazim-kosei* are lacking in *A. pseudoflavum*. The campanulate perigone and ovoid capsule (3.9–4 × 3–3.5 mm) of *A. kazim-kosei* contrast with the elliptical-campanulate perigone and globose capsule (3–3.5 × 3–3.5 mm) of *A. pseudoflavum*. Finally, the seeds of *A. kazim-kosei* (c. 2.8–3 × 1.5–2.5 mm) are smaller and irregularly angled compared to the larger, rectangular-rounded seeds (2.5–3 × 1.5–2 mm) of *A. pseudoflavum*.

*A. kazim-kosei* seeds are elliptic-oval, relatively larger (2.8–3.5 × 1.5–2.0 mm). Testa ornamentation regularly papillate, papillae dome-shaped (height 15–20 µm, width 20–30 µm). Anticlinal walls raised and distinct; periclinal walls convex. *A. pseudoflavum* seeds smaller (2.5–3.0 × 1.5–1.8 mm), with denser, smaller papillae (height 10–15 µm, width 15–25 µm). Papillae closely packed, surface finely textured. *A. kazim-kosei* pollen grains trizonocolpate, prolate-elliptic, P 42–47 µm, E 23–27 µm. Exine microreticulate, lumina 0.5–1.0 µm, muri moderately thick. *A. pseudoflavum* pollen grains slightly smaller (P 38–43 µm, E 21–25 µm), lumina smaller (0.3–0.8 µm), muri thicker and denser.

The phylogenetic tree built from nuclear ITS molecular data has strongly supported the delimitation of new species. The Maximum Likelihood (ML) tree has been constructed based on data from 28 taxa, which has a total of 660 aligned positions (Figure 6). The trees are well-resolved and contain 28 ingroup taxa from sect. *Codonoprasum* in addition to one outgroup (*A. ursinum*). The resulting tree topology provides multiple distinct clades that correlate to both geographic and morphological groups within the section. The ML tree indicates that *A. kazim-kosei* and *A. pseudoflavum* are sister taxa positioned adjacent to each other, but they are distinctly separated from each other. The bootstrap support value of 100% for the clade defined by *A. kazim-kosei* and 98% for the clade defined by *A. pseudoflavum* provides additional strong statistical evidence for the differentiation of these two phylogenetic groups [10].

All other taxa included in Figure 6 from sect. *Codonoprasum* form several distinct and well-supported clades. These clades include a clade that contains *A. flavum*, *A. gilanense*, *A. lenkoranicum* and *A. melanantherum*; a clade that contains *A. kunthianum* and *A. rupestre*; a clade that contains *A. parciflorum*, *A. stamatiae and A. castellanense*; and a clade that contains *A. paniculatum* and closely related taxa. *A. kazim-kosei* does not cluster with any of these clades and, therefore, represents an independent evolutionary lineage.

Our findings support the phylogeny presented by Duchoslav et al. [10] for species in the genus *Allium*, specifically sect. *Codonoprasum* in which five major clades (I–V) consisting of 48 taxa and three genomic loci were defined. Clade II is east Mediterranean species; *A. pseudostamineum*, *A. tauricola* and *A. rupestre*. The evidence suggests that *A. kazim-kosei* is an early diverging member belonging to a geographically restricted group of species from the eastern Mediterranean and Irano–Turanian regions.

In addition to that, Appendix A Table A1 provides further evidence for the uniqueness of *A. kazim-kosei* as compared to other taxa within sect. *Codonoprasum*, the genetic divergence values using the Kimura 2-parameter method (K2P), between *A. kazim-kosei* and the morphologically closest relative, *A. pseudoflavum* is 0.0854 (8.54%) and the closest genetic relative to *A. kazim-kosei* based on K2P genetic distance is *A. pseudostamineum* (0.0896; 8.96%). The genetic divergence values for *A. kazim-kosei* relative to other species in sect. *Codonoprasum* range from 0.0896 (vs. *A. pseudostamineum*) to 0.1639 (vs. *A. lenkoranicum*). All of these values of distance exceeds the reported average range of interspecific divergence within sect. *Codonoprasum* (0.5–5.0%) [11,15,16]. Moreover, the genetic distance between *A. kazim-kosei* and *A. pseudoflavum* (8.54%) is approximately 10 times higher than the average distance observed among closely related congeneric species within the section. These data provide strong evidence that *A. kazim-kosei* is genetically distinct from all other analyzed species [15].

A key finding was the complete congruence of the morphological and micromorphological characters, in conjunction with the molecular phylogenies (both ITS and *trnL*), indicating that this multi-source evidence of morphological and molecular phylogenetics is viewed as the gold standard in taxonomically difficult genera, such as *Allium* [1,3,4,6]. Conforming to the same species limit with four independent datasets of nuclear ITS, chloroplast *trnL*, morphology, and SEM micromorphology gives a high level of confidence for the recognized species [9,15].

The occurrence of *A. kazim-kosei* is particularly noteworthy, being restricted only to gypseous soils, as edaphic specialization is a known driver of speciation in many taxa, including *Allium* [24,38]. Gypsum soils form achievable isolative areas, which can lead to allopatric or peripatric speciation [14,20]. The narrow endemic distribution of *A. kazim-kosei* is in stark contrast to the more widespread distribution of *A. pseudoflavum*, which occurs in steppic and rocky habitats in the Central and Eastern Anatolia [5,25]. The restricted ranges of both species and the genetic and morphological differentiation indicate that *A. kazim-kosei* probably originated via peripatric speciation following the isolation of a population on a specialized gypsum substrate, which warrants further investigation using population genomic data [1,11]. The Endangered (EN) conservation status reflects the extreme rarity of this species and the need for immediate conservation actions to help in its recovery [11,14].

The addition of *A. kazim-kosei* to the increasing number of Anatolian Allium species highlights the high biodiversity of the region [14,16,20,21]. To illustrate this point, all of the recently described species are members of the *Codonoprasum* section. Many new species have been described in this section, highlighting its complexity and the necessity for additional fieldwork in regions of Türkiye that have received little attention [11,12,13].

## 5. Conclusions

In summary, the combined results from morphological, micromorphological, and molecular phylogenetic analyses provide strong multi-faceted evidence supporting *A. kazim-kosei* as a distinct new species in *Allium* sect. *Codonoprasum*. Morphologically, *A. kazim****-****kosei* is readily distinguished from its closest relative, *A. pseudoflavum*, by several diagnostic characters, including bulb size and tunic color, leaf structure, spathe venation, inflorescence and pedicel dimensions, tepal morphology, presence of interstaminal teeth, capsule shape, and seed size. Micromorphologically, SEM observations reveal consistent differences in seed testa papillae and pollen exine ornamentation between the two species. Molecular phylogenetic analyses based on ITS and *trnL* sequences place *A. kazim-kosei* as a well-supported sister lineage to *A. pseudoflavum* (bootstrap support 100% and 98%, respectively). The K2P genetic distance between the two species (8.54% for ITS) is substantially higher than typical interspecific divergences within sect. *Codonoprasum*. The new species is known only from three localities on gypsum soils in Central Anatolia, with an AOO of 8 km^2^ and EOO of 45 km^2^. Based on IUCN criteria, it is assessed as Endangered (EN) [B1ab(ii,iii) + B2ab(ii,iii)]. This discovery increases the number of known *Allium* species in Türkiye to 239, and the number of taxa in sect. *Codonoprasum* to 74. Finally, this study demonstrates the value of an integrative taxonomic approach combining morphology, micromorphology, and molecular data for species delimitation in taxonomically challenging groups, and highlights the importance of continued botanical exploration in Anatolia’s specialized edaphic habitats.

## Figures and Tables

**Table 1:** A comparison of selected characters differing among *Allium kazim-kosei* and *A. pseudoflavum* (data from [25]).

	*Allium kazim-kosei*	*A. pseudoflavum*
Bulb	1–1.2 × 0.4–0.6 cm	1.5–2 × 0.5–1.5 cm
Outer tunics	creamy-white to light brownish, 0.5–1 cm neck formed on the stem	grayish-brown, 1.5–4 cm neck formed on the stem
Inner tunic	brownish	yellowish-white
Scape	12–20 cm × 1 mm	20–50 cm × 1–2 mm
Leaves	2–3 (–4) pieces, 6–15 cm × 0.5–0.8 mm, solid, shorter than the scape	2–4 pieces, 20–30 cm × 0.5–1 mm, hollow, equal to scape
Spathe valves	unequal, 2 times the length of the flower	unequal, 2–4 times the length of the flower
Longer valves	4–4.5 (–10) cm, with 3–5 veins	7–12 cm, with 8–10 veins
Shorter valves	2.5–3.5 cm, with 2–5 veins	5–7 cm, with 8–10 veins
Inflorescence	2–2.5 cm in diameter, 7–18 flowered	2.5–4 cm in diameter, 10–25 flowered
Pedicels	0.5–1.2 cm	1.5–2.5 cm
Perigone	campanulate, 4–4.5 × 3.5–4 mm, pale yellow, sometimes with brown markings on the upper half, with prominent green veins	elliptical-campanulate, 4–6 × 4.5–5.5 mm, pale yellow, greenish yellow
Tepals	equal, elliptic to ovate mucronate, 4.2–4.5 × 1.8–2 mm; outer tepals 4.2–4.5 × 1.8–2 mm, boat-shaped, upper edges of inner tepals curved inwards	unequal, elliptic-oblong, inner tepals 3.5–4 (–5) × 1–1.5 mm, mucronate at apex, slightly recurved; outer tepals 4–4.5 (–5) × 1.5–2 mm, acute tip, sometimes recurved mucronate
Filaments	off-white, 4.5–5.5 mm	yellow, 5–5.5 mm
Interstaminal teeth	with short interstaminal teeth	absent
Anthers	yellow-cream, c. 1 mm	yellow, 0.5–1 mm
Ovary	narrowly globose, 1.5–1.8 × 1.5–1.9 mm; surface reticulate, margins clearly tuberculous	globose, 1.5–2 × 2–2.5 mm, surface tuberculous
Style	5–5.5 mm	6–7 mm
Capsule	ovoid, c. 3.9–4 × 3–3.5 mm	globose, 3–3.5 × 3–3.5 mm
Capsule valves	ovoid, 2.5–3.5 × 3–3.5 mm	valves obovate 4.75–5 × 4.25–4.75 mm
Seed	c. 2.8–3 × 1.5–2.5 mm	3.5–4 × 2–2.5 mm

**Table 2:** Comparative Seed and Pollen.

Character	*Allium kazim-kosei*	*Allium pseudoflavum*
Seed size (mm)	2.8–3 × 1.5–2.5	3.5–4 × 2–2.5
Papilla height (µm)	15–20	10–15
Papilla width (µm)	20–30	15–25
Papilla arrangement	Regularly papillate	Dense, compact
Pollen size (P × E, µm)	42–47 × 23–27	38–43 × 21–25
Exine ornamentation	Microreticulate, larger lumina (0.5–1)	Microreticulate, smaller lumina (0.3–0.8)
Muri	Moderately thick	Thicker, more pronounced

## Data Availability

The original contributions presented in this study are included in the article. Further inquiries can be directed to the corresponding author.

## Supplementary Materials

The following supporting information can be downloaded at: https://www.mdpi.com/article/10.3390/life16050852/s1, Table S1: excel spreadsheet.

## Appendix A

### Appendix A.1. Kimura 2-Parameter (K2P) Genetic Distances Based on ITS Sequences

**Table A1:** Dataset: 14 nucleotide sequences, 558 aligned positions. Analyses were conducted using the Kimura 2-parameter model [17] with pairwise deletion in MEGA 12 [48]. Values below the diagonal represent the number of base substitutions per site.

No	Taxon	1	2	3	4	5	6	7	8	9	10	11	12	13	14	15
1	*A. kazim-kosei*	-														
2	*A. pseudoflavum*	0.0854	-													
3	*A. pseudostamineum*	0.0896	0.0149	-												
4	*A. tauricola*	0.1031	0.0329	0.0331	-											
5	*A. rupestre (PX999857)*	0.1115	0.0391	0.0351	0.0436	-										
6	*A. kunthianum*	0.1145	0.0468	0.0460	0.0415	0.0020	-									
7	*A. rupestre*	0.1228	0.0543	0.0558	0.0522	0.0161	0.0200	-								
8	*A. flavum*	0.1402	0.0812	0.0836	0.0710	0.0775	0.0836	0.0987	-							
9	*A. parciflorum*	0.1497	0.0813	0.0838	0.0735	0.0685	0.0749	0.0811	0.0575	-						
10	*A. paniculatum*	0.1544	0.0898	0.0834	0.0684	0.0590	0.0703	0.0766	0.0699	0.0614	-					
11	*A. gilanense*	0.1544	0.0834	0.0814	0.0802	0.0706	0.0770	0.0876	0.0451	0.0410	0.0614	-				
12	*A. stamatiae*	0.1519	0.0875	0.0833	0.0827	0.0659	0.0767	0.0873	0.0531	0.0511	0.0551	0.0309	-			
13	*A. lenkoranicum*	0.1639	0.0920	0.0901	0.0871	0.0795	0.0856	0.0940	0.0532	0.0470	0.0633	0.0076	0.0348	-		
14	*A. turcicum subsp. turcicum*	0.1567	0.1028	0.1031	0.0986	0.0862	0.0962	0.0981	0.0802	0.0717	0.0694	0.0781	0.0653	0.0801	-	
15	*A. ursinum (outgroup)*	0.4483	0.4228	0.4752	0.3904	0.3744	0.3876	0.4029	0.3788	0.3567	0.3611	0.3532	0.3596	0.3592	0.3542	-

Note: The full ITS distance matrix including all 28 taxa is available from the corresponding author upon request or in the Supplementary Materials (Table S1). Only the 15 most phylogenetically relevant taxa (14 ingroup species + outgroup) are shown here for brevity, focusing on the position of *A. kazim-kosei* within sect. *Codonoprasum*.

### Appendix A.2. Taxon Sampling, Collection Localities, Voucher Information, and GenBank Accession Numbers for Allium Species Analyzed in This Study

**Table A2:** Collection localities, voucher specimens, and GenBank accession numbers of *Allium* taxa analyzed for ITS regions.

Species	Access Number	Sect.	Locality
*A. kazim-kosei*	PZ344465	*Codonoprasum*	Turkey, Günyüzü district of Eskişehir province, around Kavuncu village, 39.40830° N, 31.90574° E, gypseous soils, 886 m a.s.l., 12 June 2025, M. Balos 5650.
*A. pseudoflavum*	PZ344466	*Codonoprasum*	Turkey, Şanlıurfa, Karaköprü, Dağeteği (Germuş) village, Germuş mountains, northern slopes, 37°12′51.68″ K, 38°50′59.14″ D, rocky areas, 760 m., 30.05.2025, M. Balos 5713
*A. turcicum* subsp. *fusciflorum*	PZ344468	*Codonoprasum*	(Mardin: Türkiye) Mardin Province, Derik region, GAP valley, rocky slopes, 37°21′46.90″ K, 40°17′54.88″ D, 940 m, 07.05.2025, M. Balos 5668 (HUEH).
*A. yilandaghense*	PZ344469	*Codonoprasum*	(Batman: Türkiye) Batman, Sason, Sason mountains, between the villages of Çalışırlar and Taşyuva, 38°22′11.55″ K, 41°31′31.39″ D, 2215 m., hills, step, 14.06.2025, M. Balos 5715.
*A. turcicum* subsp. *turcicum*	PZ344470	*Codonoprasum*	(Batman: Türkiye) Batman, Sason, Sason mountains, between the villages of Çalışırlar and Taşyuva, 38°22′21.10″ K, 41°29′23.61″ D, 1494 m., roadside, step area, 14.06.2025, M. Balos 5714.
*A. euphariticum*	PZ344471	*Codonoprasum*	(Elazığ:Türkiye) Elazığ Province, Harput district, Serince village, roadside, steppe area, 1270 m a.s.l., 22.06.2023, M. Balos 5502 & V. Sonay, Y. Yeşildal (holotype HARRAN, isotype HARRAN).
*A. parciflorum*	KP221829.1	*Codonoprasum*	
*A. gilanense*	MT303159.1	*Codonoprasum*	
*A. paniculatum*	MK776928.1	*Codonoprasum*	
*A. stamatiadae*	OR116805.1	*Codonoprasum*	
*A. marginatum*	OR116810.1	*Codonoprasum*	
*A. fuscum*	PP834289.1	*Codonoprasum*	
*A. oleraceum*	FJ664286.1	*Codonoprasum*	
*A. goumenissanum*	OR116786.1	*Codonoprasum*	
*A. dentiferum*	KP221815.1	*Codonoprasum*	
*A. castellanense*	KP221825.1	*Codonoprasum*	
*A. flavum*	OM891951.1	*Codonoprasum*	
*A. melanantherum*	AJ412739.1	*Codonoprasum*	
*A. praescissum*	OM891954.1	*Codonoprasum*	
*A. lenkoranicum*	MT303162.1	*Codonoprasum*	
*A. rupestre*	PX999857.1	*Codonoprasum*	
*A. tauricola*	PX999870.1	*Codonoprasum*	
*A. daninianum*	PX999767.1	*Codonoprasum*	
*A. hermoneum*	PX999807.1	*Codonoprasum*	
*A. pseudostamineum*	PX999838.1	*Codonoprasum*	
*A. ursinum*	FR682005.1	*Arctoprasum*	
