# Correction: Köse, Y.B.; Balos, M.M. *Allium kazim-kosei*, a New Species (*A*. sect. *Codonoprasum*, Amaryllidaceae) from Central Anatolia (Türkiye). *Life* 2026, *16*, 852

**DOI:** 10.3390/life16071161

**Published:** 2026-07-14

**Authors:** Yavuz Bülent Köse, Mehmet Maruf Balos

**Affiliations:** 1Department of Pharmaceutical Botany, Faculty of Pharmacy, Anadolu University, 26470 Eskişehir, Türkiye; 2Department of Pharmaceutical Botany, Faculty of Pharmacy, Harran University, 63050 Şanlıurfa, Türkiye; mbalos@gmail.com

## References

In the “References” section of our published manuscript [1], the title and metadata (author names, journal title, year, and volume/page numbers) of Reference [8] were incorrectly cited due to an inadvertent typographical oversight during production. The correct spelling of the source is given below.
8.Bednorz, L.; Krzymińska-Bródka, A.; Czarna, A. Seed morphology and testa sculptures of some *Allium* L. species (Alliaceae). *Acta Agrobot.*
**2011**, *64*, 33–38. https://doi.org/10.5586/aa.2011.015.

The authors state that the scientific conclusions are unaffected. This correction was approved by the Academic Editor. The original publication has also been updated.
